# Social cognition in idiopathic focal dystonia: preliminary evidence on its relationship with cognitive and affective-behavioral functioning

**DOI:** 10.1007/s10072-026-09110-4

**Published:** 2026-05-19

**Authors:** Alfonsina D’Iorio, Francesco Ferraiuolo, Andrea Barbaro, Gianluca Scotto di Tella, Assunta Trinchillo, Francesco Habetswallner, Gabriella Santangelo, Marcello Esposito

**Affiliations:** 1 Department of Psychology, University of Campania “Luigi Vanvitelli”, Viale Ellittico, 31, Caserta, 81100 Italy; 2 St George’s, University of London, London, UK; 3 Clinical Neurophysiology Unit, Cardarelli Hospital, Naples, Italy

**Keywords:** Idiopathic focal dystonia, Non-motor symptoms, Social cognition, Theory of mind, Empathy, Apathy

## Abstract

**Introduction:**

Although motor symptoms are the defining feature of dystonia, increasing evidence indicates that non-motor symptoms substantially affect patients’ quality of life. Among these, social cognition remains insufficiently explored, particularly in relation to global cognitive functioning and affective-behavioral dimensions.

**Methods:**

In this study, 25 patients diagnosed with Idiopathic Focal Dystonia (IFD) and 25 healthy controls (HCs) underwent assessments of social cognition (cognitive and affective Theory of Mind [ToM] and empathy), global cognitive functioning (MoCA), depression (BDI) and apathy (DAS).

**Results:**

Patients diagnosed with IFD performed significantly worse than HCs on cognitive and affective ToM tasks, whereas no group differences were observed for the remaining measures. Within the IFD group, cognitive ToM performance correlated with global cognitive functioning, and affective ToM correlated with apathy but not with depression.

**Conclusion:**

These findings demonstrate specific impairments in social cognition and suggest links between ToM deficits, cognitive dysfunction and motivational disturbance. By identifying a pattern of social cognition deficit in IFD patients, this study advances understanding of its non-motor symptoms and underscores the need for further research on its impact on daily functioning and quality of life.

## Introduction

Dystonia is a movement disorder characterized by sustained, involuntary muscle contractions that result in repetitive movements or abnormal postures. It may occur as an isolated condition or in association with other neurological or systemic disorders. The original classification [1] distinguishes between primary and secondary dystonia. Primary dystonia is defined by the absence of other neurological abnormalities, apart from the movement disorder itself, and often has a genetic basis. In contrast, secondary dystonia has been linked to brain lesions, metabolic diseases, or other genetic conditions. A more recent framework [2] classifies dystonia based on multiple factors, including body distribution, age of onset, temporal pattern, family history, phenomenology and the presence of additional clinical features. According to this classification, body distribution may be focal, segmental, multifocal, hemidystonia or generalized. In focal dystonia, only one body region (upper face, lower face, neck, larynx, upper limbs, trunk, lower limbs) is affected.

Traditionally, dystonia has been regarded purely as a motor disorder. However, recent studies have highlighted the presence of non-motor symptoms that can significantly impact patients’ quality of life. Among these, the most relevant include neuropsychiatric symptoms, sleep disturbances, sensory deficits and cognitive impairments [3, 4]. These symptoms are not only secondary consequences of motor dysfunction but may represent an integral part of the disease itself [5].

The clinical relevance of non-motor symptoms is particularly evident in their impact on quality of life [6]. Psychological distress, cognitive deficits and impairments in social cognition appear to be key determinants of patients’ well-being, whereas motor symptoms seem to have a lesser influence [7]. Although many studies have documented psychiatric and cognitive alterations in dystonia patients, findings on cognitive functioning remain inconsistent, highlighting the need for further investigations to identify potential specific endophenotypes [8].

Movement disorders have been widely associated with deficits in social cognition, defined as the ability to perceive and interpret others’ mental states [9]. However, research specifically addressing social cognition in dystonia remains limited. Social cognition encompasses several processes, including Theory of Mind (ToM) and empathy. These constructs are closely related, but rely on partially overlapping, yet distinct, neural circuits [10, 11]. ToM refers to the ability to attribute mental states, beliefs and emotions to others [12] and can be divided into cognitive and affective components [13]. Cognitive ToM involves understanding others’ thoughts, beliefs and intentions, whereas affective ToM refers to the ability to infer others’ emotions. ToM deficits have been observed across several neurological conditions [14–17], including dystonia [18, 19]. Empathy, the ability to recognize, share and respond to others’ emotional states [20], is relatively under-investigated in dystonia. Current evidence on its relationship with dystonia is limited and inconclusive [18, 21].

Deficits in social cognition may occur as primary disorder or as a consequence of other impairments [22]. Therefore, it is crucial to examine their associations with broader cognitive functioning and mood alterations. In dystonia, previous studies have reported a link between social cognition and cognitive deficits, particularly in executive functions [18, 23], as well as associations with depressive symptoms [21, 24] and reduced quality of life [25]. However, the overall impact of social cognitive impairments in dystonia remains insufficiently understood and needs further investigation [26].

Considering this evidence, the present study aims to assess social cognition functioning, specifically empathy and both the cognitive and affective dimensions of ToM, and to examine their relationship with global cognitive functioning and behavioral aspects in patients with idiopathic focal dystonia. We hypothesize that social cognitive deficits are an intrinsic feature of idiopathic focal dystonia. Therefore, we expect patients to perform significantly worse than healthy controls on ToM tasks. Additionally, we seek to explore the relationship between social cognition and both cognitive and behavioral functioning.

In addition to addressing these aims, the present study provides a comprehensive and multimodal assessment of social cognition in IFD by combining multiple tasks targeting both cognitive and affective components of Theory of Mind, along with self-reported empathy. Furthermore, by including a heterogeneous sample of focal dystonia phenotypes, this study aims to offer a broader characterization of social cognitive functioning beyond the more frequently investigated cervical dystonia. Finally, the integration of cognitive, affective, and behavioral measures allows for a more comprehensive understanding of the potential interplay between these domains.

## Materials and methods

### Participants

Consecutive outpatients with Idiopathic Focal Dystonia (IFD) were recruited from February 2022 to October 2023 at the Neurology Department of “Antonio Cardarelli” Hospital in Naples, according to the following inclusion criteria: (1) diagnosis of IFD in accordance with the clinical diagnostic criteria [2]; (2) absence of cognitive decline, defined by an age- and education-adjusted score on the Italian version of the Montreal Cognitive Assessment (MoCA) [27, 28] greater than 15.5; (3) absence of current or past psychiatric and/or neurological disorders according to the DSM-5 diagnostic criteria [29]. Subsequently, healthy subjects (HCs) were enrolled to match with patients for demographics features. These participants were recruited among patients’ friends and university center employees. To be included in the study, subjects had to meet the same inclusion criteria as the IFD group, with the exception of the first one.

For each participant, demographic data (i.e. gender, age, years of schooling) were recorded. All participants provided written informed consent prior to the start of the study, which was approved by the local ethics committee in accordance with the 1964 Declaration of Helsinki.

### Neuropsychological evaluation

#### Cognitive functioning

In order to ensure participant eligibility and sample homogeneity, global cognitive functioning was assessed using the Italian version of the Montreal Cognitive Assessment (MoCA) [27, 28], a brief screening tool sensitive to mild cognitive impairment across multiple domains. Additionally, language comprehension was evaluated using the Token Test [30].

#### Social cognition

All participants underwent an assessment of both cognitive and affective ToM. Task selection was guided by the need to provide a comprehensive exploration of the multifaceted aspects of mental state attribution across different modalities and levels of ecological validity. Affective ToM was measured using the Reading The Mind in the Eyes Task (RMET) [31] and the Emotion Attribution Task (EAT) [32]. The RMET was included as a well-established measure of affective mental state recognition, sensitive to subtle social-cognitive alterations, whereas the EAT provides a more contextually rich and verbally mediated assessment of emotion inference. Cognitive ToM was assessed using the Theory of Mind Picture Stories Task (ToM PST) [33], the Advanced Theory of Mind Test (ATM) [34], which differ in both format and inferential demands.

The ToM PST minimize linguistic load requiring to organize cartoon sequences to evaluate the understanding of intentions and causal relationships in social contexts, while the ATM demands the interpretation of verbally presented scenarios, involving complex interactions and requiring more sophisticated reasoning.

In addition, the Empathy Quotient (EQ) [35] was administered to provide a self-reported measure of trait empathy, providing complementary information on empathic tendencies that are conceptually related yet distinct from performance-based ToM abilities.

In details, the RMET consists of 37 black-and-white items (1 practice item and 36 test stimuli), each depicting a cropped image of a person’s eyes, accompanied by four adjective options. The stimuli include young, middle-aged, and older adults of all genders. Participants are asked to select the adjective that best represents the emotion conveyed by the depicted gaze.

The EAT requires participants to infer the emotional state of the protagonist based on a short story read aloud by the examiner. The 35 stories depict emotionally charged situations in which the protagonist may experience emotions such as sadness, anger, embarrassment, disgust, happiness, jealousy, or envy.

The ToM PST requires participants to rearrange a sequence of four cartoon panels displaying various social interactions. If the sequence is incorrect, the examiner arranges the images in the correct order before proceeding to follow-up questions. These questions assess the participant’s understanding of the scenario, the emotional states of the characters, and their motivations.

The ATM consists of 13 short narratives describing social interactions between two or more characters. After each story, the participant is asked to explain the reasoning behind specific characters’ actions, aiming to assess their ability to infer intentions, beliefs, and underlying social cues.

The EQ is intended to measure how easily you pick up on other people’s feelings and how strongly you are affected by other people’s feelings. It’s a 60-item self-report questionnaire comprising 40 empathy items and 20 filler items, rated on a 4-point Likert scale: “strongly agree”, “slightly agree”, “slightly disagree”, “strongly disagree”. The scoring system assigns two points to “strongly agree” and one point to “slightly agree”, whereas for the other half it assigns two points to “strongly agree” and one point to “slightly disagree”; the remaining response options and the filler items score 0.

#### Depression and apathy

Depression and apathy were evaluated using the Beck Depression Inventory-II (BDI-II) [36] and the Dimensional Apathy Scale (DAS) [37], two standardized self-report questionnaires.

The BDI–II is designed to assess the presence and severity of depressive symptoms in adolescents and adults. It comprises 21 items, each rated on a 4-point Likert scale (0–3) reflecting increasing levels of symptom severity.

The DAS assess the multidimensional aspects of apathy in clinical and non-clinical populations. It consists of 24 items rated on a 4-point Likert scale, designed to capture three distinct subdomains of apathy: Executive, Emotional, and Initiation. Unlike unidimensional measures, the DAS allows for a more nuanced characterization of apathetic symptoms, distinguishing between deficits in goal-directed behavior, emotional reactivity, and cognitive initiation.

### Statistical analysis

A comparison between IFD and HCs groups was carried out using the two-tailed Mann-Whitney U test for the analysis of demographic, cognitive and behavioral variables. For each comparison, the effect size r was also calculated to quantify the magnitude of the effect; the r values were interpreted based on conventional thresholds, where values around 0.10 indicate a small effect, around 0.30 a medium effect, and 0.50 or higher a large effect.

In order to examine the association between ToM scores with cognitive and behavioral variables within the IFD group, Spearman’s correlation analysis was performed.

Given the exploratory nature of the study and the relatively large number of statistical tests performed, analyses were initially conducted without correction for multiple comparisons. However, to control for potential Type I error inflation, a False Discovery Rate (FDR) correction using the Benjamini–Hochberg procedure was applied as a sensitivity analysis.

In addition, a post hoc sensitivity power analysis was conducted to estimate the minimum detectable effect size given the sample size (*n* = 25 per group), assuming a two-tailed alpha level of 0.05 and 80% statistical power.

The critical alpha level for all analyses was set at < 0.05. All statistical analyses were conducted using IBM SPSS Statistics version 29.

## Results

Twenty-five IFD patients (18 females) were enrolled, classified according to the affected body region [2] as follows: 12 patients with cervical dystonia (CD), 9 patients with blepharospasm (BSP), 2 patients oro-mandibular dystonia (OMD), 1 patient with lower limb dystonia, 1 with focal hand dystonia. All patients received botulinum toxin injections as treatment for focal dystonia. Additionally, none of the participants were chronically under benzodiazepine.

Furthermore, a group of 25 healthy subjects was enrolled.

### Comparison between groups

No significant differences were observed between the groups in demographic variables (i.e., age, years of schooling) or in affective measures (i.e., BDI-II, DAS).

Regarding ToM performance, IFD patients showed significantly lower scores on the EAT and the ToM PST, with medium effect sizes. After applying FDR correction for multiple comparisons, none of the observed group differences remained statistically significant. No significant differences were found between groups on RMET, ATM and EQ scores. (Table 1).

**Table 1:** Comparison between IFD and HCs groups on demographic, cognitive and behavioral variables

	IFD(M ± SD)	HCs(M ± SD)	U	r	*p*	p(FDR)
Number of subjects	25 (f=18)	25 (f=18)	-	-	-	
Age (years)	59.76 ± 12.57 (26–79)	58.88 ± 13.05 (24–77)	301.5	0.03	0.831	
Education (years)	11.64 ± 3.73 (5–17)	10.96 ± 3.54 (5–17)	280	0.09	0.506	
MoCA	23.48 ± 3.38	24.16 ± 2.73	295.5	0.04	0.740	0.833
Token test	32.19 ± 1.74	32.61 ± 2.47	119.5	0.11	0.418	0.537
RMET	22.04 ± 4.89	24.16 ± 4.52	231	0.22	0.112	0.252
ToM PST	41.92 ± 9.25	47.76 ± 5.63	194	0.32	0.021*	0.103
ATM	9.41 ± 2.28	9.12 ± 2.54	250.5	0.07	0.599	0.674
EAT	23 ± 4.34	26.16 ± 4.33	195.5	0.32	0.023*	0.103
EQ	49.04 ± 9.58	49.87 ± 9.78	265.5	0.03	0.823	0.823
BDI-II	10.24 ± 9.62	11.64 ± 7.26	248	0.17	0.210	0.378
DAS	22.58 ± 7.91	20.32 ± 9.93	251	0.13	0.326	0.489

*p* < 0.05

Abbreviations: *MoCA* montreal cognitive assessment, *RMET* reading the mind in the eyes task, *ToM PST* theory of mind picture stories task, *ATM* advanced theory of mind test, *EAT* Emotion attribution task, *EQ* empathic quotient, *BDI-II* beck depression inventory-II, *DAS* dimensional apathy scale

### Correlation between ToM and empathy scores and cognitive variables

A significant correlation was observed between MoCA scores and ToM PST performance in the IFD group (Table 2), suggesting that higher global cognitive functioning is associated with better cognitive ToM abilities.

**Table 2:** Correlation between ToM and empathy measures and cognitive variables in IFD patients

	RMET	ToM PST	ATM	EAT	EQ
	rho (*p* value)	rho (*p* value)	rho (*p* value)	rho (*p* value)	rho (*p* value)
MoCA	0.013 (0.950)	0.52 (0.0077)*	0.001 (0.997)	-0.233 (0.262)	0.304 (0.149)
Token test	0.489 (0.090)	0.251 (0.409)	0.345 (0.272)	0.434 (0.139)	0.142 (0.642)

*, < 0.05

Abbreviations: *MoCA* montreal cognitive assessment, *RMET* reading the mind in the eyes task, *ToM PST* theory of mind picture stories task, *ATM* advanced theory of mind test, *EAT* emotion attribution task, *EQ* empathic quotient

### Correlation between ToM and empathy scores and behavioral variables

In the IFD group, a significant correlation emerged between RMET performance and DAS scores (Table 3), indicating that greater levels of apathy are related to increased difficulties in affective ToM.

**Table 3:** Correlation between ToM and empathy measures and behavioral variables in IFD patients

	RMET	ToM PST	ATM	EAT	EQ
	rho (*p* value)	rho (*p* value)	rho (*p* value)	rho (*p* value)	rho (*p* value)
BDI-II	-0.146 (0.485)	0.178 (0.395)	-0.196 (0.382)	0.25 (0.229)	-0.240 (0.259)
DAS	-0.491 (0.015)*	0.148 (0.49)	-0.306 (0.177)	-0.16 (0.456)	-0.201 (0.357)

*, < 0.05

Abbreviations: *MoCA* montreal cognitive assessment, *RMET* reading the mind in the eyes task, *ToM PST* theory of mind picture stories task, *ATM* advanced theory of mind test, *EAT* emotion attribution task, *EQ* Empathic Quotient, *BDI-II* beck depression inventory-II, *DAS* dimensional apathy scale

## Discussion

The present study investigated social cognition in patients with idiopathic focal dystonia, with a focus on empathy and Theory of Mind, by comparing their performance with that of healthy subjects. The aim was to determine the presence of social cognition impairments and to clarify whether these may represent a common feature across different clinical presentation of dystonia.

Our findings suggest that socio-cognitive functioning in IFD is not globally compromised, but shows a selective pattern of impairment. Patients performed worse than controls on the EAT and on the ToM PST, whereas no significant group differences emerged on the RMET or the ATM. This discrepancy likely reflects differences in task demands, as these measures engage in distinct components of affective and cognitive ToM and may vary in their sensitivity to subtle deficits.

Importantly, these findings should be interpreted with caution. After applying False Discovery Rate (FDR) correction for multiple comparisons, none of the observed group differences remained statistically significant. This indicates that the present results should be considered exploratory rather than confirmatory. Nevertheless, the presence of medium effect sizes for selected measures (EAT and ToM PST) suggests potentially meaningful trends that may reflect subtle alterations in higher-order social cognitive processes and may inform future hypothesis-driven studies.

With regard to affective ToM, the results point to a selective difficulty in inferring emotional states from verbally mediated information. While RMET performance was comparable between groups, IFD patients showed lower scores on the EAT, particularly for more complex emotions such as disgust and envy. This divergence may be explained by the different cognitive requirements of the two tasks. The RMET focuses primarily on the visual recognition of subtle facial cues, whereas the EAT requires the integration of contextual and semantic information, which may make it more sensitive to detecting impairment. This pattern is consistent with previous findings in dystonia [19, 23, 41], which reported preserved RMET performance and a reduced performance in the EAT, supporting the notion that basic aspects of affective mentalizing may remain relatively intact. Further support comes from studies using facial emotion recognition tasks [24, 26], which failed to detect significant differences between patients and controls. Taken together, these findings suggest a selective disruption affecting more complex components of affective social cognition in IFD.

In addition, an interesting finding is the association that was observed between RMET performance and apathy within the IFD group. Although no between-group differences emerged on this task, lower RMET scores were associated with higher levels of apathy, suggesting that motivational factors may influence the efficiency of emotion recognition. While this relationship between apathy and ToM has received limited attention in dystonia, similar associations have been reported in other movement disorders, including Parkinson’s disease [15]. One possible explanation is that apathy in IFD patients, potentially linked to dysfunction within basal ganglia circuits, may lead to reduced attention and responsivity to socially relevant cues. This aspect deserves further investigation, as it may contribute to better understand the variability in ToM performance observed within this population.

The analysis of cognitive ToM also revealed differences in performance across tasks. Patients with IFD showed significantly poorer performance than healthy controls on the ToM PST, whereas no group differences emerged on the ATM. Although both tasks assess cognitive ToM, they differ in their demands. The ToM PST places greater emphasis on false belief reasoning, while the ATM primarily involves the interpretation of verbally presented social scenarios. Given that false belief processing represents a more complex component of ToM [19], the selective impairment observed on the ToM PST may reflect its higher cognitive demands. This interpretation is supported by the association between ToM PST performance and global cognitive functioning, as measured by the MoCA, within the IFD group. These findings suggest that more demanding ToM processes may rely on general cognitive resources, as reported in other populations [38, 39].

When compared to the existing literature, the present findings contribute to a heterogeneous pattern of results. In contrast with the ATM findings reported here, Lagravinese et al. [19] observed lower ATM scores in patients with cervical dystonia compared to healthy controls, particularly in those with tremor, and found a correlation between ATM performance and MoCA scores. Differences in sample characteristics, such as the inclusion of multiple dystonia subtypes in the present study, may partially explain this discrepancy. On the other hand, studies using a faux pas task in cervical dystonia [23; 42] did not find significant differences between patients and controls, in line observed on the ATM. However, the evidence remains mixed, as other studies employing the same kind of measure reported impaired performance in cervical dystonia patients compared to controls [18].Overall, these findings suggest that cognitive ToM deficits in IFD may not be consistently detectable across tasks, but are more likely to appear in situations that place higher demands on reasoning about others’ mental states and general cognitive resources, explaining the inconsistencies observed across studies.

With regard to empathy, no significant differences emerged between IFD patients and controls. This finding is consistent with previous studies in cervical dystonia [18, 26]. Interestingly, in contrast to results observed by Ellement et al. [21], no association was observed between EQ scores and depressive symptom in our study, suggesting a relative independence of these constructs. One possible explanation for this discrepancy might again be related to sample heterogeneity, as previous studies have focused primarily on cervical dystonia. Supporting this interpretation, a study by Berman et al. [40] showed that different focal dystonia phenotypes are associated with distinct patterns of altered microstructure within key regions of the basal ganglia and cerebellar circuits. Given the involvement of multiple neural substrates implicated in both motor and cognitive functions, these structural differences may contribute to the variability in cognitive deficits across various focal dystonia subtypes, potentially accounting for some of the observed heterogeneity in clinical and cognitive profiles.

Importantly, the present results carry significant clinical implications. While the motor symptoms of dystonia have been extensively studied and addressed, the current findings underscore the presence of non-motor and affective impairments that may considerably affect patients’ quality of life [6–8]. The identification of social cognitive deficits highlights the need to broaden clinical assessment protocols to include measures of Theory of Mind (ToM). Moreover, rehabilitative interventions specifically targeting social cognition may enhance functional adaptation and psychosocial well-being in individuals with idiopathic focal dystonia (IFD).

By examining ToM across a heterogeneous IFD population, this study contributes to a more comprehensive understanding of whether social cognitive impairments may represent a shared feature across different focal dystonia subtypes. Indeed, in contrast to most previous investigations, which have predominantly focused on cervical dystonia [18, 19, 21, 23–26], the present study includes patients with multiple IFD phenotypes. This broader approach allows for a more global characterization of social cognitive functioning within the IFD spectrum, although it also introduces additional variability. It should also be noted that social cognition comprises multiple processes that were not directly assessed. Although the present study focused on ToM and empathy due to their central role in social functioning and their extensive investigation in the literature [19, 21, 23, 41, 42], other components of social cognition may also be relevant for a comprehensive understanding of social functioning.

The absence of significant group differences in RMET, ATM, and EQ performance also warrants careful consideration. These findings may suggest a relative preservation of specific components of social cognition in IFD. However, alternative explanations should be considered. It is possible that these tasks are not sufficiently sensitive to detect subtle impairments in this population, particularly given differences in task complexity and the specific cognitive processes involved. Additionally, the limited sample size may have reduced the statistical power to identify more subtle group differences. Therefore, these null findings should be interpreted cautiously and cannot be taken as definitive evidence of intact functioning.

Some limitations should be acknowledged. First, the relatively small sample size limits the statistical power of the study. A sensitivity power analysis indicated that the study was adequately powered to detect large effects (Cohen’s d ≈ 0.80), whereas the observed effects for ToM PST and EAT (d ≈ 0.68) were associated with moderate statistical power (approximately 55–60%). Smaller effects were substantially underpowered, suggesting that non-significant findings may reflect insufficient statistical sensitivity rather than the absence of true differences.

Second, a relatively large number of statistical tests were conducted. Although a False Discovery Rate (FDR) correction was applied as a sensitivity analysis, none of the findings remained statistically significant after correction. This further supports the exploratory nature of the study and highlights the need for cautious interpretation.

Methodological limitations should also be considered. The MoCA was used as a screening tool to exclude global cognitive impairment and ensure sample homogeneity; however, it does not provide a detailed evaluation of specific cognitive domains. In particular, the lack of dedicated measures of executive functions limits the possibility of fully investigating their contribution to ToM performance, which has been highlighted in previous literature. The absence of quality of life measures, who could helped clarify how cognitive, affective and social factors influence overall functioning, also restricts the interpretation of the functional impact of the observed alterations. Furthermore, the use of self-report measures introduces the possibility of response bias. Clinical variables, that may have influenced the social cognition performance, such as disease duration, severity and pharmacological treatment, were not systematically investigated. Finally, the lack of neuroimaging data restricts the ability to draw direct inferences regarding the neural substrates underlying ToM performance in this population.

Future studies should address these limitations by including larger and more homogeneous samples, conducting a priori power analyses, and employing comprehensive neuropsychological batteries with a focus on executive functions. The integration of neuroimaging and longitudinal designs will also be essential to better characterize the nature and progression of social cognitive alterations in dystonia.

Despite its exploratory nature, the present study contributes to the literature in several ways. By adopting a multidimensional approach to social cognition and including a heterogeneous IFD sample, it provides a broader perspective on socio-cognitive functioning in dystonia. Moreover, the integration of cognitive and behavioral correlates offers preliminary insights into the mechanisms underlying variability in ToM performance. Although these findings require replication in larger samples, they help identify potentially relevant patterns that may guide future research.

In conclusion, the present findings should be considered preliminary and suggest a potential pattern of social cognitive alterations in patients with IFD, particularly affecting higher-order components of Theory of Mind. These alterations appear to be associated with apathy and global cognitive functioning, highlighting the need for further investigation in larger, adequately powered, hypothesis-driven studies.

## Data Availability

Data and materials for the experiments are available from the corresponding author upon reasonable request.
